# Modelling cognitive affective biases in major depressive disorder using rodents

**DOI:** 10.1111/bph.12603

**Published:** 2014-07-01

**Authors:** Claire A Hales, Sarah A Stuart, Michael H Anderson, Emma S J Robinson

**Affiliations:** School of Physiology and Pharmacology, University of Bristol, University WalkBristol, UK

## Abstract

Major depressive disorder (MDD) affects more than 10% of the population, although our understanding of the underlying aetiology of the disease and how antidepressant drugs act to remediate symptoms is limited. Major obstacles include the lack of availability of good animal models that replicate aspects of the phenotype and tests to assay depression-like behaviour in non-human species. To date, research in rodents has been dominated by two types of assays designed to test for depression-like behaviour: behavioural despair tests, such as the forced swim test, and measures of anhedonia, such as the sucrose preference test. These tests have shown relatively good predictive validity in terms of antidepressant efficacy, but have limited translational validity. Recent developments in clinical research have revealed that cognitive affective biases (CABs) are a key feature of MDD. Through the development of neuropsychological tests to provide objective measures of CAB in humans, we have the opportunity to use ‘reverse translation’ to develop and evaluate whether similar methods are suitable for research into MDD using animals. The first example of this approach was reported in 2004 where rodents in a putative negative affective state were shown to exhibit pessimistic choices in a judgement bias task. Subsequent work in both judgement bias tests and a novel affective bias task suggest that these types of assay may provide translational methods for studying MDD using animals. This review considers recent work in this area and the pharmacological and translational validity of these new animal models of CABs.

**Linked Articles:**

This article is part of a themed section on Animal Models in Psychiatry Research. To view the other articles in this section visit http://dx.doi.org/10.1111/bph.2014.171.issue-20

## Introduction

Antidepressant drugs were first discovered serendipitously in the 1950s when clinicians observed that patients treated with certain antihistamines (Kuhn, 1958) or drugs developed to treat tuberculosis showed improved mood (Delay *et al*., 1953; see Slattery *et al*., 2004). The discovery of these drugs provided a route to developing animal models to predict efficacy of novel antidepressants resulting in the first ‘animal models of depression’: reserpine-induced behavioural deficits (Askew, 1963) and immobility in the forced swim test (FST; Porsolt *et al*., 1977). The latter test of immobility, which is interpreted as a measure of depression-like behavioural despair, has become the most widely used assay to study major depressive disorder (MDD) in rodents, with subsequent refinements to the method including the modified FST (Detke *et al*., 1995), which can detect efficacy mediated through noradrenergic versus serotonergic mechanisms, as well as the tail suspension test being used as a murine model (Steru *et al*., 1985; see Cryan *et al*., 2005). Although not the main focus of this review and discussed in detail elsewhere, selectively in-bred and genetic strains have been developed to study depression-like phenotypes, as well as approaches to induce this phenotype in normal rodents (Willner, 1984; 2005; Nestler *et al*., 2002; Cryan and Holmes, 2005; Henn and Vollmayr, 2005; Rygula *et al*., 2005; Cryan and Slattery, 2007; Nestler and Hyman, 2010; Pollak *et al*., 2010; Neumann *et al*., 2011; Schmidt *et al*., 2011; Overstreet, 2012). The best characterized of these approaches are chronic mild stress (Willner *et al*., 1987; 1992; Willner, 1997; 2005), chronic social stress procedures (Kudryavtseva *et al*., 1991; Rygula *et al*., 2005), early life adversity procedures such as maternal separation (Matthews *et al*., 1996; see Schmidt *et al*., 2011) and olfactory bulbectomy which produces a range of behavioural and physiological changes that emulate those seen in MDD (Leonard, 1984; Kelly *et al*., 1997).

Developing and validating animal models for depression research is particularly challenging but is an area of scientific need. Recently, there has been a move towards establishing translational methods for research into MDD, although the nature of this disease presents challenges for emulating aspects of the disorder in non-human species (see Berton *et al*., 2012 for a recent discussion). The human psychiatric condition is a heterogeneous disorder manifesting as a mixture of emotional, behavioural and somatic symptoms. The DSM-5 criteria for diagnosis of MDD requires the presence of at least one of two core symptoms, low mood and/or anhedonia, and at least five other symptoms from a list that includes psychomotor retardation, suicidal ideation, sleep changes and weight changes (American Psychiatric Association, 2013). Often, the diagnosis of MDD is based on questionnaires and clinical interviews where the patient’s subjective reporting of symptoms is used to establish a diagnosis. The subjective nature of this clinical assessment represents a major challenge when working with non-human species.

Cognitive impairments in MDD have long been known to exist and recent developments in objective measures of this aspect of the disorder have provided new opportunities for developing novel methods for use in rodents. While a range of different cognitive impairments have been reported in MDD (for detailed discussion see Austin *et al*., 2001; Elliott *et al*., 2011; Roiser and Sahakian, 2013), this review focuses on cognitive affective biases (CABs). CAB is a term used in psychology and cognitive neuroscience to describe how cognitive processes are influenced by emotion. Negative CABs have been reported to co-occur with depressive symptoms across a range of cognitive domains including perception, attention and learning and memory (see Mathews and MacLeod, 2005; Clark *et al*., 2009; Gotlib and Joormann, 2010). These findings are summarized in Table 1. Clinical studies have shown that depressed patients are more likely to remember negative rather than positive emotional information in self-relevant tasks and interpret key social signals, such as facial expressions of emotion, as either more negative or less positive than healthy volunteers (Gur *et al*., 1992; Bouhuys *et al*., 1999; Surguladze *et al*., 2004). Similar negative biases have also been linked to increased risk of relapse (Bouhuys *et al*., 1999) and tend to persist into clinical remission (Hayward *et al*., 2005). Increased negative versus positive emotional interpretation has also been associated with vulnerability to depression (Hayward *et al*., 2005; Chan *et al*., 2007; Joormann *et al*., 2007; Dearing and Gotlib, 2009).

**Table 1:** Summary of findings from human neuropsychological tests of cognitive affective biases in MDD

Bias	Test	Observations in depressed patients	References
Attention	Emotional Stroop (Identify the colour of emotional words while ignoring the meaning)	↑ Latency for negative words	Gotlib and McCann, 1984; Segal *et al*., 1995; Broomfield *et al*., 2007
		↑ Perigenual ACC response to negative words	McCabe and Gotlib, 1995; Mitterschiffthaler *et al*., 2003
	Dot probe task (Respond to the location of a dot that replaces an emotional stimulus)	↑ Response latency for positive versus negative stimuli	Mathews *et al*., 1996; Gotlib *et al*., 2004; Joormann and Gotlib, 2007
	Affective go/no-go (Respond/withhold response to emotional stimuli)	↑ Omission errors to positive stimuli	Murphy *et al*., 1999; Elliott *et al*., 2002; Erickson *et al*., 2005; Kyte *et al*., 2005; Kaplan *et al*., 2006
		↑ Subgenual cingulate response to negative stimuli	
Perception	Emotional categorization (categorizing the valence of affective stimuli e.g. self-referent phrases or facial expressions	↓ Response latency to negative versus positive faces	Gilboa-Schechtman *et al*., 2002; Joormann and Gotlib, 2006; Harmer *et al*., 2009b; Murphy *et al*., 2009; Yoon *et al*., 2009
		↑ Amygdala response to negative faces	Sheline *et al*., 2001; Fales *et al*., 2009
Memory	Emotional recall (recall of emotionally valenced words)	↓ Recall of positive versus negative stimuli	Gilboa-Schechtman *et al*., 2002; Ellwart *et al*., 2003; Harmer *et al*., 2009b
		↑ Amygdala response to recalled negative stimuli	Hamilton and Gotlib, 2008
Feedback sensitivity	Probabilistic reversal learning	↑ Reversal following negative feedback	Elliott *et al*., 1997; Murphy *et al*., 2003
		↓ Dorsal ACC response to negative feedback	Steele *et al*., 2007
		↑ Amygdala response to negative feedback compared with controls	Taylor Tavares *et al*., 2008

## The current status of animal models of depression

The limitations associated with animal models of depression have been discussed extensively (Willner, 1984; 2005; Nestler *et al*., 2002; Cryan and Holmes, 2005; Cryan and Slattery, 2007; Nestler and Hyman, 2010; Pollak *et al*., 2010; Berton *et al*., 2012; O’Leary and Cryan, 2013) and therefore will not be considered here beyond a brief summary of the issues relevant to this review. When considering animal models of depression, the term ‘model’ is often used to describe both methods to induce a depression-like phenotype and those methods used to assay depression-like behaviour. The differentiation between a ‘model’ and a ‘test’ is important as the ‘model’ is often dependent on ‘tests’ of depression-like behaviour to validate the phenotype. While a number of more general behavioural features can be measured in rodents which may relate to depression, for example, poor coat condition, body weight changes and aggression (Cryan and Holmes, 2005; Cryan and Slattery, 2007; Hendrie *et al*., 2013), assays which are specific to depression-like behaviours are still limited. The two key areas tested in animals are behavioural despair and anhedonia. Assays of behavioural despair are the FST and tail suspension test where immobility time, swimming and/or climbing behaviours are measured (see Cryan and Slattery, 2007). Initially developed and validated using prototypical monoaminergic antidepressant drugs, these tests have been criticized as having limited validity for non-monoaminergic targets (Berton and Nestler, 2006). Locomotor-related changes in immobility may also give false positives in these tests (Porsolt *et al*., 1977; Slattery and Cryan, 2012). Anhedonia is perhaps a more straightforward feature to measure in rodents as a number of approaches, including the sucrose preference test and intracranial self-stimulation, can be used to measure hedonic responses and deficits are sensitive to antidepressant drugs (Vogel *et al*., 1986; Willner *et al*., 1987; Zacharko and Anisman, 1991). However, anhedonia is only one symptom seen in MDD and exists in a number of other disease states including schizophrenia (see Wolf, 2006) and Parkinson’s disease (see Loas *et al*., 2012). Therefore, measuring anhedonia only may not be the best approach when considering the wider spectrum of symptoms seen in MDD.

The major problems with the current assays used in preclinical studies are limited translational validity and the risk of identifying false positives. The issues with animal models of depression also extend to safety pharmacology and the ability to predict drugs which increase the risk of psychiatric side effects, especially suicidal ideation and behaviour, early in the development process (Gibbons and Mann, 2011). Failures associated with poor efficacy, for example, the neurokinin NK_1_ receptor antagonist and failed antidepressant, aprepitant (Keller *et al*., 2006), or unacceptable neuropsychiatric side effect profiles, for example, the cannabinoid CB_1_ receptor antagonist/inverse agonist, rimonabant (Griebel *et al*., 2005; Topol *et al*., 2010), have significant cost implications for the pharmaceutical industry, as well as associated negative impacts on patients. New methods for identifying novel drug targets and predicting antidepressant drug efficacy or pro-depressant risks are therefore needed.

## Cognitive biases in mood disorders

In 1967, Beck hypothesized that early adverse life experiences lead to the development of negative schemata that ultimately cause negative biases in the processing of emotional information (Beck, 1967; 1976). More recently, several reviews have converged on a theory that these cognitive impairments may contribute to the development, maintenance and treatment of depression (Robinson and Sahakian, 2008; Clark *et al*., 2009; Harmer *et al*., 2009a; Elliott *et al*., 2011; Roiser *et al*., 2012).

Perhaps the most important evidence in support of a cognitive neuropsychological mechanism in MDD comes from recent studies where acute treatments with antidepressant drugs were shown to induce positive biases in emotional processing without any subjective effects on mood (Harmer *et al*., 2009b; Harmer, 2013). This work underpins a cognitive neuropsychological model of depression and antidepressant action, which posits that negative affective biases associated with MDD are caused by alterations in monoamine transmission, with these alterations being effected by a combination of environmental and/or genetic factors (Harmer, 2008; 2013; Harmer *et al*., 2009a; Pringle *et al*., 2011; Harmer and Cowen, 2013). Over time this biased input causes the automatic processing of affective information to be shifted more negatively, creating stable self-reinforcing negative schemata. In addition, these negative schemata may themselves instantiate negative biases to maintain the depressive state. Importantly, this model contradicts traditional models of antidepressant action in suggesting that antidepressants alter the processing of affective stimuli, rather than affecting mood directly (Robinson and Sahakian, 2008; Clark *et al*., 2009; Harmer *et al*., 2009a). These behavioural findings have been echoed in studies using functional magnetic resonance imaging where remediation of altered activity within emotional processing circuits has been linked to antidepressant efficacy (Leppänen, 2006; Ressler and Mayberg, 2007; Victor *et al*., 2013).

A key outcome of this work is the potential to develop translational methods for preclinical research using reverse translation, whereby similar neuropsychological processes are evaluated in non-human species. Development and validation of methods suitable for studies in animals are also important for testing this cognitive neuropsychological hypothesis of depression and antidepressant pharmacology.

## Cognitive affective bias in animals: overview

Considerable progress has been made in terms of characterizing CABs associated with negative affect and MDD in humans. However, only recently have these processes been explored in animals. Paul *et al*. (2005) proposed that while most of the cognitive outputs of emotion studied in humans involve language-based tasks, many could, with appropriate modifications, be studied in animals. Brain imaging studies suggest that in patients, negative CABs and processing of emotional information involves neural circuits which are conserved across species (Cryan and Holmes, 2005). The challenge is therefore to develop methods to study these CABs in animals using non-language-based approaches while preserving the same underlying neurobiological processes.

## Ambiguous cue interpretation and judgement bias

The first study investigating CAB in animals was carried out by Harding *et al*. (2004) who showed that manipulating affective state using chronic mild stress induced a negative or ‘pessimistic’ bias in the way rats responded to ambiguous stimuli. Rats were first trained to discriminate between two distinct tone cues: one predicting reward and another predicting punishment. In order to probe CABs, rats were then presented with intermediate ambiguous tone cues. During ambiguous cue presentation, anticipation of reward (lever approach) was interpreted as a positive bias while anticipation of punishment (withhold response) was interpreted as representing a negative bias. Induction of a putative negative affective state using a mild stress procedure resulted in rats making fewer reward responses to ambiguous ‘probe’ tones, indicating decreased anticipation of a positive outcome. This pessimistic judgement bias is comparable with findings in depressed patients (Wright and Bower, 1992). This initial study highlighted the potential of this approach for depression- and welfare-related research, although the original format of the task involved potential confounds. Animals in this task were trained to make an active response to obtain reward but to refrain from responding to avoid punishment, leading to potential motivational and locomotor confounds. To address this, other groups have sought to modify this original methodology from a go/no-go task to a go/go task where an active response to obtaining reward and avoiding punishment are initially trained.

Enkel *et al*. (2010) were the first to report a go/go version of this CAB task. In this modified task, rats were trained to make an active lever press response to either obtain reward or avoid a mild electric shock. In this model, a pharmacological induction of a stress-like state using the co-administration of the noradrenaline reuptake inhibitor reboxetine (although see later discussion), and the glucocorticoid corticosterone, resulted in a negative judgement bias. This was seen by a reduction in reward responses without a corresponding increase in avoidance responses. Additionally, in a congenital learned helpless rat strain performing the same task, a shift in responding away from obtaining reward and towards increased avoidance of punishment was observed (Enkel *et al*., 2010). These results suggest that subtle differences exist in the way judgement bias is expressed in animals, which may be relevant to different types of negative affective state (for discussion, see Mendl *et al*., 2010b). Similar results were also obtained for this task when animals were exposed to chronic social stress (Papciak *et al*., 2013), replicating the original study by Harding *et al*. (2004) using this go/go methodology. Furthermore, the same group have also used the ambiguous cue interpretation approach to show that animals’ negative CAB in this task is associated with their individual stress reactivity and anhedonia (Rygula *et al*., 2013). In terms of the relationship between affective state and cognitive bias, these data suggest that negative affective states in rats and vulnerability to stress-induced anhedonia are associated with pessimistic behaviour in judgement bias tasks. Conversely, it has also been shown in rats that induction of a positive affective state through tickling, measured by the production of 50-kHz ultrasonic vocalizations that are proposed to be akin to human laughter, causes a positive CAB (Rygula *et al*., 2012).

Pharmacological evaluation of judgement bias using this type of reward versus punishment ambiguous cue interpretation task is limited to a single study testing antidepressant drugs (Anderson *et al*., 2013). In this study, acute treatment with fluoxetine and diazepam failed to have any effects, while reboxetine treatment reduced anticipation of reward, similar to the effects seen by Enkel *et al*. (2010). The effects seen with reboxetine are contrary to the predicted antidepressant effects of the drug and are observed with (Anderson *et al*., 2013) or without co-administration of corticosterone (Enkel *et al*., 2010). It is not clear why these effects occur and studies of CAB in humans have found positive biases following similar acute treatments (Harmer *et al*., 2003). Using chronic administration of fluoxetine, a tendency towards a positive shift in judgement bias was seen; although as this was not a robust finding, further studies are needed to assess the predictive validity of using this approach to evaluate antidepressant drugs. A summary of these studies investigating CAB in rodents is given in Table 2.

**Table 2:** Summary of judgement bias studies investigating CABs in rodents and humans

Species	Cue	Response	Manipulation to alter affective state	Key result	Reference
Rat	Auditory tone	Go/No-go (lever press)	Unpredictable housing	Slower and fewer responses to ‘reward’ tones	Harding *et al*., 2004
Rat	Auditory tone	Active choice	Unpredictable housing	No effect	Parker, 2008
			Environmental enrichment		
Rat	Auditory tone	Active choice	Congenital learned helplessness rats	Decreased positive and increase negative responses to ambiguous tones	Enkel *et al*., 2010
			Drug-induced negative affective state	Decreased positive responses to ambiguous tones	
Rat	Auditory tone	Active choice	Acute and chronic antidepressant treatments	Chronic but not acute treatments reduced negative bias	Anderson *et al*., 2013
Rat	Auditory tone	Active choice	Baseline vulnerability and chronic stress	Chronic stress increased negative bias and is associated with baseline vulnerability	Rygula *et al*., 2013
Rat	Auditory tone	Active choice	Chronic social stress	Increased negative bias following chronic social defeat stress	Papciak *et al*., 2013
Rat	Auditory tone	Active choice	Tickling	Increased expectation of reward in tickled rats	Rygula *et al*., 2012
Rat	Spatial location	Go/No-go (locomotor)	Environmental enrichment	Faster responses to the probe nearest the unrewarded location	Burman *et al*., 2008
Rat	Spatial location	Go/No-go (locomotor)	Bright light	Slower responses to all ambiguous locations	Burman *et al*., 2009
Rat	Spatial location	Go/No-go (locomotor)	Congenital learned helplessness rats	Slower response latencies to ambiguous stimuli	Richter *et al*., 2012
			Environmental enrichment	Increased speed of responding to ambiguous stimuli in both congenital learned helpless and control rat lines	
Rat	Spatial location	Go/No-go (locomotor)	Environmental enrichment	Increased number of optimistic responses in animals transferred from unenriched to enriched cages	Brydges *et al*., 2011
Rat	Spatial location	Go/No-go (locomotor)	Juvenile stress	Increased number of optimistic choices in animals subjected to juvenile stress	Brydges *et al*., 2012
Rat	Cued spatial location	Go/No-go (locomotor)	Adolescent chronic mild stress	Induction of negative bias	Chaby *et al*., 2013
Mouse	Olfactory cues	Go/No-go (locomotor)	High versus low anxiety strains	High anxiety strain show negative bias under stress condition	Boleij *et al*., 2012
Man	Ambiguous and unambiguous predictor stimuli	Latency to decide	High versus low Positive and Negative Affect Schedule (PANAS) mood inventory scores	Bias towards expecting hazards as opposed to rewards	Paul *et al*., 2011
Man	Auditory tone	Go/Go	Healthy volunteers	Correlation between negative bias and anxiety	Anderson *et al*., 2012
Man	Auditory tone	Go/Go	Healthy volunteers	Correlation between negative bias and rumination scores	Schick *et al*., 2013

### Spatial judgement bias methodology

An alternative approach to the operant lever press judgement bias task that has been developed is the spatial judgement bias task, where two different food outcomes (either rewarding vs. aversive or high reward vs. low/no reward) are initially associated with a specific spatial location. Judgement bias is then evaluated by measuring the latency to approach both the reference locations and intermediate positions. A negative bias in this version of the task is seen when the animal makes a slower response to an intermediate position, indicating a reduced anticipation of finding the reward. Where an aversive food outcome such as quinine-treated food is used, the slowed responding is proposed to show an expectation that the intermediate location is associated with the aversive outcome. Using this approach, Richter *et al*. (2012) showed that as with the operant judgement bias task (Enkel *et al*., 2010), a congenital learned helplessness rat strain exhibited a pessimistic judgement bias. After being transferred to enriched cages to promote a positive affective state, rats were retested and found to show a more optimistic judgement bias (Richter *et al*., 2012). In another spatial judgement task, rats were trained to expect food from a goal pot in one location but no food from a goal pot in another location (Burman *et al*., 2008). Environmental enrichment was removed from half the animals during training and prior to testing. It was found that a judgement bias existed in response to the ambiguous locations that were nearest the unrewarded location. Similarly, a more negative judgement of ambiguous spatial stimuli has been reported in response to acute induction of a negative state through illumination of test apparatus with bright light, although this occurred only in rats that had been trained in low lighting levels then tested in bright light (Burman *et al*., 2009). Another version of the spatial judgement bias task has been conducted using two positively valenced outcomes – a high value reward and low value reward, where the reward outcome was paired with texture of the floor (coarse or fine sandpaper) on the approach to either outcome (Brydges *et al*., 2011). Animals that had been housed with environmental enrichment showed optimistic judgment biases to ambiguously textured approaches. The same format of spatial judgement bias task was used to show that rats exposed to chronic mild stress during adolescence displayed a more negative judgement bias than rats that experienced predictable conditions (Chaby *et al*., 2013); although surprisingly, it has also been reported that rats exposed to juvenile stress showed increased optimistic choices (Brydges *et al*., 2012). However, the authors do discuss the caveat that in this study animals may have been using an optimal foraging strategy and hence have been more willing to take a risk on the high food reward as they had low body weight (Brydges *et al*., 2012). Table 2 provides a summary of findings in rodents using spatial judgement bias tasks.

Only one judgement bias study has been reported for mice where odour cues were used to predict rewarding or aversive food outcomes (Boleij *et al*., 2012; Table 2). Latencies were recorded for responses to an ambiguous odour involving a mixture of the two reference cues to assess judgement bias, and strain differences relating to anxiety were shown to be associated with pessimistic decisions (Boleij *et al*., 2012).

### Judgement bias in other species

Although the focus of this review is CAB in rodents, it is interesting to note that this type of methodology has been applied in a number of different species [e.g. dogs (Casey *et al*., 2008; Mendl *et al*., 2010a; Burman *et al*., 2011; Müller *et al*., 2012), pigs (Douglas *et al*., 2012), sheep (Doyle *et al*., 2010a,b; 2011a,b; Verbeek *et al*., 2014), grizzly bears (Keen *et al*., 2013); rhesus macaques (Bethell *et al*., 2012), chicks (Salmeto *et al*., 2011; Hymel and Sufka, 2012), starlings (Bateson and Matheson, 2007; Matheson *et al*., 2008; Brilot *et al*., 2010) and honeybees (Bateson *et al*., 2011)]. The methods employed have varied somewhat, but include go/no-go tasks and spatial judgement bias tasks using high versus low rewards or high versus no rewards. Overall, these studies suggest CAB assessed using this approach can be observed across a wide range of species from primates (Bethell *et al*., 2012) to invertebrates (Bateson *et al*., 2011). The observation that honeybees show pessimistic judgements in a go/no-go model raises some issues about how much this behaviour is related to any conscious experience of affective state (see Mendl *et al*., 2011 for a discussion).

In order to test the translational validity of this type of task, studies in humans have also been carried out (Paul *et al*., 2011; Anderson *et al*., 2012; Schick *et al*., 2013; Table 2). These studies used healthy volunteer populations and either tone (Anderson *et al*., 2012; Schick *et al*., 2013) or image based (Paul *et al*., 2011) judgement bias tasks where participants first learnt to associate distinct cues with obtaining reward or avoiding punishment. Responses to intermediate ambiguous cues resulted in a similar profile of responding to that seen in rodents and, in each study, the participant’s judgement bias scores correlated with questionnaire measures of mood (Paul *et al*., 2011; Anderson *et al*., 2012; Schick *et al*., 2013). Using this type of strategy may help to further validate the methodology being used in animals, although studies in patient populations are still to be reported.

### Neural circuits relevant to CAB

**Figure 1:**
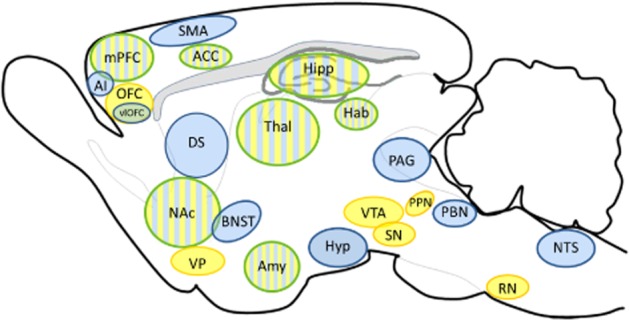
Neural circuits relevant to cognitive affective biases. This schematic diagram of a rat brain illustrates the similarities and differences between key brain areas linked to reward-related neural circuits versus those which regulate aversion or punishment. Measurement of CAB in the ambiguous cue interpretation task is based on identifying biases towards reward or towards avoidance of punishment, and therefore these circuits are likely to be important in this task. Areas shown in yellow have been linked to reward while those in blue are known to play a role in aversion. Some regions of the brain are involved in mediating both reward and aversion and are shown in blue/yellow with green outline. Abbreviations: ACC, anterior cingulate cortex; AI, anterior insula; Amy, amygdala; BNST, bed nucleus of the stria terminalis; DS, dorsal striatum; Hab, lateral habenular; Hipp, hippocampus; Hyp, hypothalamus; mPFC, medial prefrontal cortex; NAc, nucleus accumbens; NTS, nucleus of the solitary tract; OFC, orbitofrontal cortex; PAG, periaqueductal gray; PBN, parabrachial nucleus; PPN, pedunculopontine nucleus; RN, raphe nucleus; SMA, secondary motor area; SN, substantia nigra; Thal, thalamus; VTA, ventral tegmental area; vlOFC, ventral lateral orbitofrontal cortex; VP, ventral pallidum.

The neural circuits underlying CABs have not been well studied, but it is known that patients with MDD process positively and negatively valenced stimuli and events differently, exhibiting increased sensitivity to punishment (Kasch *et al*., 2002) and reduced sensitivity to reward (Henriques *et al*., 1994; Gotlib and Joormann, 2010). Neuroimaging studies suggest that these differences are due to dysfunction in different networks of brain regions (Eshel and Roiser, 2010; McCabe *et al*., 2012; Robinson *et al*., 2012). As CAB tasks measure biases in processing towards reward or towards avoidance of punishment, changes in these different networks involved in reward and punishment processing are likely to be important. Research has been conducted investigating the core brain areas that are involved in processing both rewarding and aversive stimuli. Human imaging studies and anatomical, pharmacological and behavioural animal experiments have identified a complex reward circuit (see Haber and Knutson, 2010) that is centred around the dopaminergic neurons of the nucleus accumbens, ventral tegmental area and substantia nigra, but also incorporates a broader cortical-basal ganglia circuit which includes the ventral striatum, ventral pallidum, anterior cingulate cortex (ACC) and parts of the prefrontal cortex (PFC; Figure 1). Other areas that have been shown to be important in mediating reward processing include the amygdala, hippocampus, lateral habenular nucleus and thalamus, as well as specific brainstem nuclei (Figure 1). Correspondingly, there is neuroimaging and neuroanatomical evidence for a network of brain areas that regulate processing of aversion-related information (see Hayes and Northoff, 2011). Despite a degree of overlap between reward and aversion processing neural areas (for example the nucleus accumbens, amygdala, hippocampus, thalamus, lateral habenular, ACC and dorsal medial PFC; Figure 1), the functional aversion-related processing network is markedly different from the reward-processing pathway. The principal brain regions for aversion processing comprise the anterior insula, orbitofrontal cortex, amygdala and ACC, with other significant areas including the dorsal striatum, bed nucleus of the stria terminalis, hypothalamus, secondary motor area, periaqueductal gray and again some precise brainstem structures (although these are different from the reward-processing nuclei; Hayes and Northoff, 2011; Figure 1). Specific studies investigating the neural circuitry involved in CABs related to interpretation of ambiguous information are limited but studies investigating memory and attentional CABs have shown that the ACC (Mitterschiffthaler *et al*., 2003), parts of PFC, orbitofrontal cortex and cingulate cortex (Elliott *et al*., 2002) and amygdala (Hamilton and Gotlib, 2008) exhibit altered activity during these tasks in humans (Table 1). These brain regions are known to be key areas involved in processing of rewarding and aversive stimuli (Figure 1). Studying CAB in judgement bias tasks utilizing rewarding and aversive outcomes, as well as only rewarding outcomes may enable greater understanding of the relative importance of these different neural pathways in contributing to abnormal cognitive biases in MDD.

**Figure 2:**
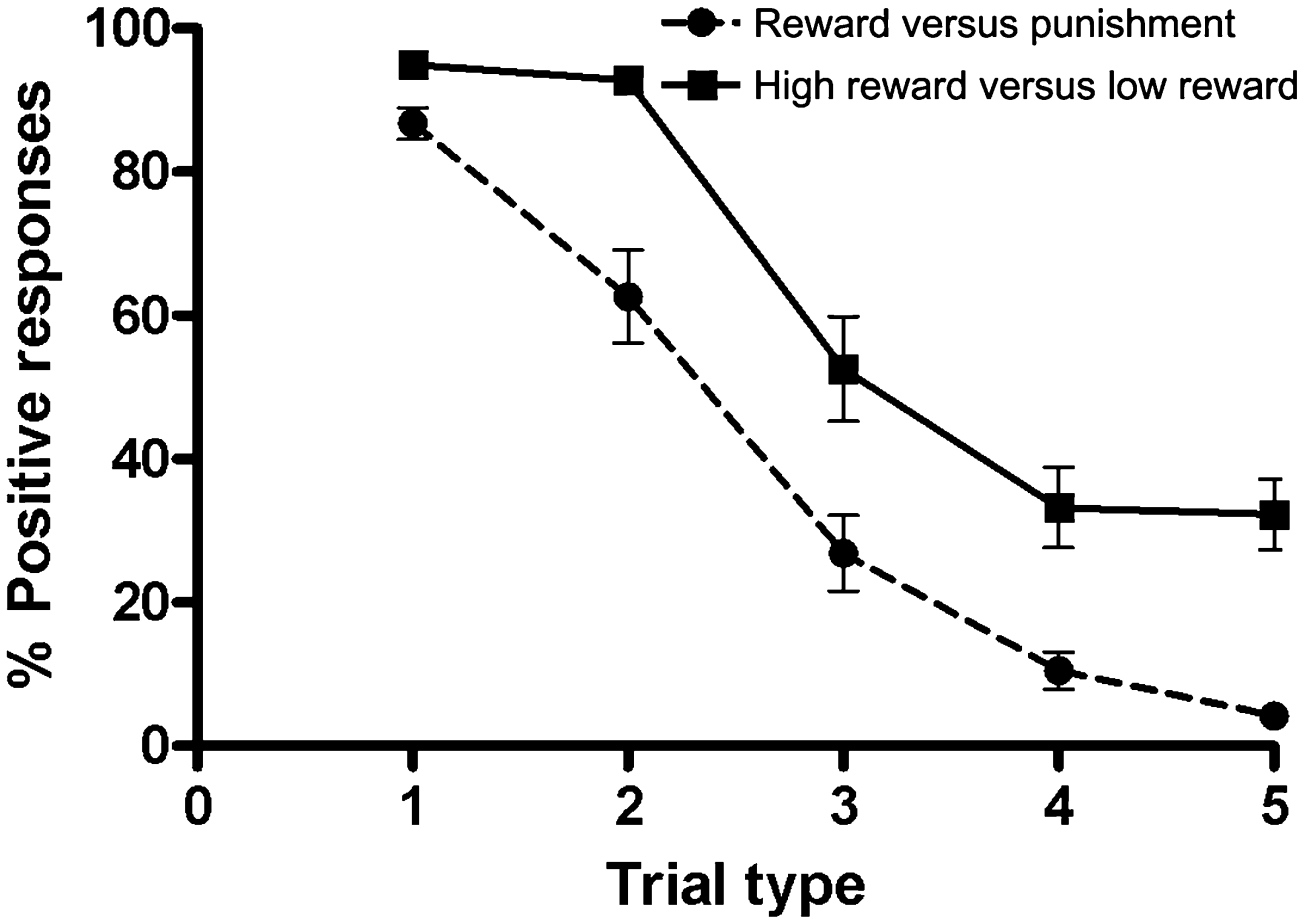
Effects of valence on performance in the operant judgement bias task. Results are shown for the percentage of positive responses made to reference and ambiguous tones for two operant judgement bias tasks. The dotted line represents the responses made to different tone cues in rats trained to respond to distinct tones and levers associated with reward (positive response) or avoidance of foot shock (data taken from Anderson *et al*., 2013; *n* = 18). The solid line represents the percentage of positive responses made to the same tone cues but in rats trained to respond for a high value (positive response) or low value reward (unpublished; *n* = 14). Tones 1–5 refer to: 1: reference positive (reward or high value reward); 2: near positive ambiguous; 3: midpoint ambiguous; 4: near negative ambiguous; 5: reference negative (avoidance of foot shock or low value reward).

We have recently piloted an operant judgement bias task which uses only reward-based outcomes (similar to the spatial judgement bias task discussed above (Brydges *et al*., 2011; Chaby *et al*., 2013) and found that the profile of responding to ambiguous cues is not the same as studies using the reward versus punishment avoidance approach (Figure 2). In our studies, rats performing the reward versus avoidance of punishment task exhibited a negative judgement bias under baseline conditions (Anderson *et al*., 2013; Figure 2). They were also more likely to make a pessimistic response during presentation of the near positive ambiguous cue. In contrast, animals trained to respond for a high value versus low value reward were less likely to anticipate the less positive outcome during any of the ambiguous cues and also responded to the midpoint tone with a neutral judgement bias (Figure 2). These findings suggest that reward-based tasks may differ in the neural circuitry involved from tasks where reward and avoidance of punishment are used, although the negative bias in this version of the task could also be due to increased stress caused by the risk of receiving a foot shock punishment.

### Validity of judgement bias tasks for depression research

Judgement bias tasks have now been evaluated in a number of species including rodents, with some promising data suggesting that negative judgement biases can be measured in animals in putative negative affective states (Mendl *et al*., 2006; 2010b). Unfortunately, pharmacological validation is limited and no studies have yet been published where the underlying neural and neurochemical mediators of these biases have been investigated in animals. There are also some caveats that exist with current methodologies which require further investigation, including the use of active choice versus go/no-go tasks, reward only outcomes versus reward and avoidance of punishment and whether ambiguous probe trials were reinforced versus non-reinforced. The use of go/go tasks helps to reduce potential confounds associated with motivation although these types of task are more challenging to train and achieve stable levels of performance. As discussed above, differences in the brain circuits involved in reward and punishment-related cognition may also influence the interpretation of reward-based tasks versus those including active avoidance of punishment. In terms of the 3Rs (reduce, replace and refine), studies which employ only reward-based outcomes have important welfare implications, as they avoid the need to use aversive training methods (Mendl *et al*., 2009). In relation to the use of reinforcement for the ambiguous probe trials, there are potential issues if the probe trials are repeated on more than one occasion. If ambiguous cues are not reinforced, the animal may learn that it is not necessary to perform any response. If trials are continuously reinforced then the animals may learn these outcomes and respond based on prior experience rather than a true ambiguous cue interpretation. One option is to use continuous reinforcement for reference cues but random reinforcement for ambiguous cues to reduce any new learning but also maintain responding. Overall, the principle of judgement bias tasks and results so far suggest that this approach has translational validity and may evaluate similar CABs in animals as those measured in humans. However, predictive validity is still to be established.

## The affective bias test: cognitive affective biases in learning and memory

**Figure 3:**
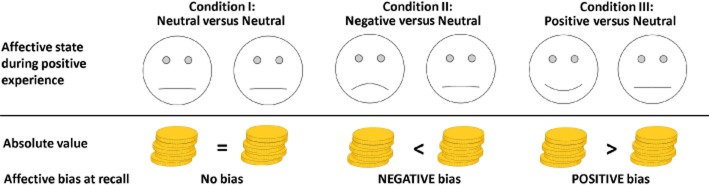
Hypothesis from which the affective bias test was derived. The assay is based on the concept that experience-dependent memory may be biased by the affective state of the animal at the time of learning. The affective bias test uses a within-subject study design where animals encounter two independent learning experiences on separate occasions. Affective state manipulations or drug treatments are paired with one of the learning experiences and cognitive affective bias is measured using a subsequent preference test.

Patients with MDD do not necessarily experience a different environment from those who do not get depressed, suggesting that it is their interaction with the environment and the effect of negative cognitive biases on learning and memory that may contribute to the development and perpetuation of the disease. In particular, studies into autobiographical memory and the phenotype of MDD suggest that the disease is associated with a propensity to recall negative information over positive information (Williams, 1992; Gotlib and Krasnoperova, 1998). Patients with MDD also demonstrate reduced experience of reward (DSM-5; American Psychiatric Association, 2013). Taken together with the cognitive neuropsychological hypothesis of antidepressant drug action, these observations suggest that memory may be biased by affective state at the time of learning. Thus, while the absolute value of a positive experience may be the same, affective state at the time of learning would modify this such that subsequent recall of that experience would be associated with a positive or negative bias (Figure 3). The affective bias test is a bowl digging task for rats (Stuart *et al*., 2013) and has been derived from this hypothesis. The task uses a within-subject study design where animals encounter two distinct learning experiences (finding a food reward in a specific digging medium) under different treatment conditions. The value of each experience is the same (receiving a single food pellet) but the animal’s affective state is manipulated prior to one experience. Affective bias is then assessed using a preference test where both reward-associated digging media are presented together and the animal’s preference for one over the other is recorded.

**Figure 4:**
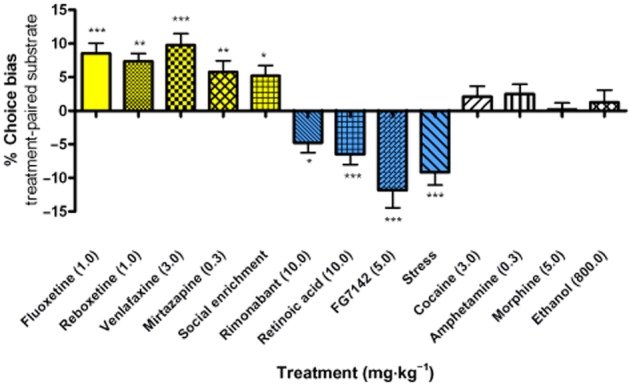
Summary of pharmacological and affective state-related validation data for the affective bias test. Acute manipulations of affective state as well as antidepressant and pro-depressant pharmacological treatments induce CABs in this rat task consistent with their effects in healthy human volunteers and patients with depression. Consistent with the hypothesis outlined in Figure 3, drugs which have antidepressant or pro-depressant effects in humans induce positive or negative biases in the affective bias task respectively. In addition, studies using stress (10 min restraint stress followed by 8 h social isolation) or environmental enrichment (8 h exposure to a highly enriched social environment) to modify affective state in the rats also induced biases consistent with their predicted effects on affective state. Yellow bars shows manipulations that caused a positive bias and blue bars indicate a negative bias. Drugs of abuse (white bars) had no effect in the test. Antidepressant drugs tested included typical and atypical drugs while pro-depressant treatments tested were the anxiogenic benzodiazepine inverse agonist, FG7142, cannabinoid CB_1_ receptor antagonist/inverse agonist, rimonabant and retinoic acid, the active ingredient of the anti-acne treatment, roaccutane. *P < 0.05, **P < 0.01, ***P < 0.001, *n =* 16 animals per group. Data in this figure are taken from Stuart *et al*., 2013.

Pharmacological and psychosocial stress manipulations were used to assess the validity of the approach (Stuart *et al*., 2013). Using a psychosocial stress manipulation, rats were found to exhibit a negative bias, making fewer choices for the experience encountered during the stress manipulations versus the experience encountered during neutral conditions (Figure 4). The opposite effect was observed when rats were exposed to a short-term environmental enrichment procedure (Figure 4) suggesting affective bias in this task is related to the rats’ affective state at the time of learning. An extensive pharmacological evaluation using acute treatments with known antidepressant and pro-depressant drugs also suggests that the method has good predictive validity (Stuart *et al*., 2013; Figure 4). Following acute treatment, both typical and atypical antidepressant drugs administered prior to learning led to a positive affective bias at recall. In terms of prodepressant manipulations, treatment with rimonabant (a CB_1_ receptor antagonist/inverse agonist), retinoic acid (the active ingredient of the anti-acne medication roaccutane) or FG7142 (a benzodiazepine inverse agonist) that caused severe anxiety in humans (Dorow *et al*., 1983) all induced a negative bias in this assay. Taken together with the effects of these treatments in human volunteers and patients, these studies suggest that the rodent affective bias test predicts both antidepressant and pro-depressant effects in man. Experiments using both stimulant and non-stimulant drugs of abuse failed to detect any biases in this assay (Stuart *et al*., 2013; Figure 4) suggesting this test is specific to depression-related behaviour and not sensitive to drugs acting directly on reward pathways.

One potential issue with this task is the possibility that the drugs or affective state manipulations have a direct effect on learning and memory or appetite which thus leads to a subsequent bias in the memory of that experience. Initial experiments suggested that effects are not associated with impairments in learning, as animals performing the task show similar rates of learning on treatment and control discrimination learning sessions. Sedative effects can also be controlled for within the protocol as latencies to approach the bowl are recorded (Stuart *et al*., 2013). Controlling for appetite effects is less straightforward although the observed profile of effects does not necessarily correspond with the drugs’ known effects on appetite (Stuart *et al*., 2013). The mechanisms involved in mediating affective bias in this test require further investigation, but initial studies evaluating the effects of drug treatment before and after learning yielded similar results, suggesting that pharmacological and affective state manipulations are acting during memory consolidation rather than acquisition (Stuart *et al*., 2013).

A possible criticism of the findings in the affective bias test is that response biases are seen after acute treatments. The field of depression has been dominated by the notion that antidepressant drugs have a delayed onset of clinical efficacy. This criticism has been applied to established assays of depression-like behaviour such as the FST and also used to support the improved validity of tests of anhedonia. However, a key issue with the delayed onset of action of antidepressant drugs is that this observation is based on the subjective reporting of mood in patients and does not necessarily mean that the acute biochemical effects of the drug are not relevant to its efficacy. In fact, the recent observation that acute treatment with antidepressant and prodepressant drugs in healthy volunteers can cause CABs (e.g. Harmer *et al*., 2003; 2004; 2009b; see Pringle *et al*., 2011) suggests that antidepressant drugs may well have acute effects on these emotional processes which then contribute to their long-term efficacy. Studies in healthy volunteers have found that emotional processing biases occurred without a subjective change in mood (Harmer *et al*., 2009b). Given the similarity between the results in healthy volunteers and the rat affective bias test, and the fact that the assay measures a neuropsychological process which is thought to contribute to MDD, this novel assay also exhibits translational validity.

Overall, the results for the affective bias task suggest this approach has good predictive and translational validity. The data obtained correspond well with similar acute drug studies carried out in healthy volunteers performing emotional recognition and characterization tasks (see Pringle *et al*., 2011). This supports the conclusion that the assay can predict both antidepressant and prodepressant pharmacology in humans.

## Other methods used to study reward and/or punishment-related behaviours

Although not necessarily defined specifically as a measure of CAB in MDD, probabilistic reversal learning tasks do offer a translational method to study how reward and punishment processing are altered in this disorder (Swainson *et al*., 2000; Paulus *et al*., 2002; Murphy *et al*., 2003; Taylor Tavares *et al*., 2008). In this task, the correct choice of stimuli has to be learnt using probabilistic trial and error feedback. Once learnt, the rule is reversed and the length of time taken to adapt responding and learn the new rule is measured. It has been shown that depressed patients performing this task made more incorrect reversals following misleading negative feedback given before the reversal occurred compared with control subjects (Murphy *et al*., 2003). This suggests that negative affective state may be linked to increased sensitivity to negative feedback in this task.

A rodent probabilistic reversal learning task has been evaluated in one study where serotonergic manipulations, including the antidepressant, citalopram, were investigated in normal rats (Bari *et al*., 2010). This study found that acute manipulations of the 5-HT system modulated sensitivity to negative feedback given after an error response, whereas chronic treatments specifically affected reward sensitivity. Another study suggested that isolation rearing of rats also altered responses to negative feedback in this same task (Amitai *et al*., 2014). Further studies using this approach are needed before the full validity of the method can be established, however, these initial findings and the translational validity of the approach are promising.

## Conclusions and future directions

The focus of this review is two behavioural approaches which have been developed to study CABs in depression using rodents. Although still in the early days of development, results so far suggest that CABs can be studied in rodents, with both decision-making during ambiguous cue interpretation and learning and memory being similarly biased by affective states in rodents and humans.

In terms of affective state induced cognitive biases, the results from judgement bias tasks provide robust and relatively well-replicated findings that animals in a putative negative affective state exhibit negative judgements when interpretation of ambiguous cues is evaluated. Results from the affective bias test also suggest that both stress-induced negative affective states and prodepressant drug treatments negatively bias reward-associated learning and memory, resulting in a relative decrease in the value attributed to a rewarding experience. These observations correspond well with data from patients with MDD (Mathews and MacLeod, 2005; Clark *et al*., 2009; Gotlib and Joormann, 2010), as well as those at risk of developing depression (Hayward *et al*., 2005; Chan *et al*., 2007; Joormann *et al*., 2007; Dearing and Gotlib, 2009). In terms of pharmacological validation, studies using the judgement bias task are limited, and it is not yet clear whether this approach provides a valid method to predict antidepressant or prodepressant pharmacology. In contrast, the affective bias test has been shown to exhibit good predictive validity, with the results obtained in rodents corresponding well with those obtained from healthy volunteer studies (Pringle *et al*., 2011; Stuart *et al*., 2013).

Pharmacological studies using these approaches are still limited and further studies in this area are needed. In terms of understanding the value of the tasks for preclinical drug development and safety pharmacology, more detailed studies using drugs with known antidepressant and prodepressant effects in humans would be of value. Pharmacological studies also offer a route to understanding more about the neural and neurochemical mediators of CABs and studies using rodents can help reveal the neurobiological processes which modulate these behaviours. This type of work has the potential to deliver a greater understanding of the relationship between CABs and MDD which may also reveal novel drug targets for improved treatments. The development of translational animal models of CABs in MDD can also be used to investigate the emerging cognitive neuropsychological hypothesis of MDD and antidepressant efficacy (Harmer, 2008; 2008; Pringle *et al*., 2011; Harmer and Cowen, 2013). Studies in animals can then be used to determine if a causal relationship exists between monoamine neurotransmitters and negative CAB in MDD and these if biases are remediated by antidepressant drugs. However, for this to be achieved, these cognitive approaches need to be more widely investigated in psychopharmacological research and drug development associated with MDD.

## Abbreviations

ACC: anterior cingulate cortex
CAB: cognitive affective bias
FST: forced swim test
MDD: major depressive disorder
PFC: prefrontal cortex
